# *Vitis vinifera* (Muscat Variety) Seed Ethanolic Extract Preserves Activity Levels of Enzymes and Histology of the Liver in Adult Male Rats with Diabetes

**DOI:** 10.1155/2015/542026

**Published:** 2015-03-17

**Authors:** Nelli Giribabu, Kilari Eswar Kumar, Somesula Swapna Rekha, Sekaran Muniandy, Naguib Salleh

**Affiliations:** ^1^Department of Physiology, Faculty of Medicine, University of Malaya, 50603 Kuala Lumpur, Malaysia; ^2^Pharmacology Division, A.U. College of Pharmaceutical Sciences, Andhra University, Visakhapatnam, Andhra Pradesh 530 003, India; ^3^Department of Zoology, Sri Venkateswara University, Tirupati, Andhra Pradesh 517502, India; ^4^Department of Molecular Medicine, Faculty of Medicine, University of Malaya, 50603 Kuala Lumpur, Malaysia

## Abstract

The effect of *V. vinifera* seeds on carbohydrate metabolizing enzymes and other enzymes of the liver in diabetes is currently unknown. We therefore investigated changes in the activity levels of these enzymes following *V. vinifera* seed extract administration to diabetic rats. *Methods*. *V. vinifera* seed ethanolic extract (250 and 500 mg/kg/day) or glibenclamide (600 *μ*g/kg/day) was administered to streptozotocin-induced male diabetic rats for 28 consecutive days. At the end of treatment, liver was harvested and activity levels of various liver enzymes were determined. Levels of thiobarbituric acid reactive substances (TBARS) were measured in liver homogenates and liver histopathological changes were observed. *Results*. *V. vinifera* seed ethanolic extract was able to prevent the decrease in ICDH, SDH, MDH, and G-6-PDH and the increase in LDH activity levels in liver homogenates. The seed extract also caused serum levels of ALT, AST, ALP, ACP, GGT, and total bilirubin to decrease while causing total proteins to increase. Additionally, the levels of ALT, AST, and TBARS in liver homogenates were decreased. Histopathological changes in the liver were reduced. *Conclusion*. Near normal activity levels of various enzymes and histology of the liver following *V. vinifera* seed ethanolic extract administration may be due to decrease in liver oxidative stress in diabetes.

## 1. Introduction 

Liver plays a central role in carbohydrate metabolism which function can be affected in diabetes [1]. Liver participates in the metabolic processes including glucose synthesis and storage [2]. The glycolytic and Krebs cycle enzymes play pivotal role in the ATP generation from glucose [3]. In glycolysis, few key liver enzymes are involved for example lactate dehydrogenase (LDH) (which converts pyruvate to lactate and* vice versa*) [4], isocitrate dehydrogenase (ICDH), *α*-ketoglutarate dehydrogenase (*α*-KDH), succinic dehydrogenase (SDH), fumarase, and malate dehydrogenase (MDH) which participate in interconversion of metabolites within the Krebs cycle [3]. Meanwhile, intermediary molecules formed in glycolytic pathway such as glucose-6-phosphate (G-6-PD) can be shunted into pentose phosphate (PPP) pathway and into the pathway that leads to glycogen or triglyceride (TG) syntheses involving 6-phospho-D-glucono-1,5-lactone. The latter process is catalyzed by glucose-6-phosphate dehydrogenase (G-6-PDH) enzyme [2]. In rats, the levels of carbohydrate metabolizing enzymes in the liver were decreased in diabetes [5].

The enzymes such as alanine aminotransferase (ALT), aspartate aminotransferase (AST), and alkaline phosphatase (ALP) which serve as biomarkers of hepatocyte damage are involved in various reactions in the liver. Plasma levels of AST and ALT were increased following hepatocyte injury while ALP, gamma glutamyl transferase (GGT), and total bilirubin levels were elevated in biliary tree obstruction [6]. Diabetes has been reported to induce pathological changes in the liver [7] such as glycogen deposition, steatosis, and nonalcoholic steatohepatitis (NAFLD) which could ultimately lead to fibrosis and cirrhosis [8]. Clinical study has revealed that type 2 diabetic patients have higher incidence of liver function test abnormalities as compared to the healthy individuals [9].

*Vitis vinifera (Linn.),* which belongs to family Vitaceae, is one of the most widely grown fruit crops in the world.* V. vinifera* possesses wide range of pharmacological activities including inhibition of platelet aggregation and low density lipoprotein (LDL) oxidation [10], antidiabetic, antioxidant [11], antimicrobial [12], and anticarcinogenesis [13]. The seed extract has been reported to protect the liver against carbon tetrachloride- (CCl_4_-) induced toxicity in rats [14]. So far, the effect of* V. vinifera* seed extract on diabetes-induced liver damage has not been fully revealed. In this study, we hypothesized that* V. vinifera* seed was capable of preserving activity levels of liver carbohydrate metabolizing enzymes and prevents liver damage in diabetes. We further hypothesized that the seed extract was able to reduce TBARS levels in the liver in diabetes. The aims of this study are therefore to investigate* V. vinifera* seed extract effect on liver carbohydrate metabolizing enzymes and other enzymes related to liver function, histopathological changes, and TBARS level in diabetes.

## 2. Materials and Methods 

### 2.1. Chemicals and Reagents

Streptozotocin (STZ) and glibenclamide were purchased from Sigma Chemicals (St. Louis, MO, USA). ALT, AST, acid phosphatase (ACP), ALP, GGT, and total bilirubin estimation kits were purchased from Randox Laboratories Ltd. (Crumlin, County Antrim, UK). Other chemicals and reagents used in this study were of analytical grade.

### 2.2. Collection and Extraction of Plant Materials

The ripe fruits of* V. vinifera* (Bangalore Blue, Muscat variety) were collected from Tirupathi, Andhra Pradesh, India, during October 2012 and authenticated by Dr. K. Madhava Chetty, Botanist, Sri Venkateswara University, Tirupati, India. The seeds were deposited in Herbarium of Department of Botany, Sri Venkateswara University with the number 86783. The seeds were separated from pulp and shade dried. The dried powdered seed materials (2 kg) were subjected to maceration in 5 L of ethanol (95%) for 3 days at room temperature and filtered into a clean round bottom flask using an adsorbent cotton wool and a filter paper (Whatman no. A-1). The whole process was repeated five times to ensure maximum yield of ethanol soluble compounds from the seed powder. The combined ethanolic extract was concentrated at 40–50°C using a rotary evaporator (Rotavapor R-210, Buchi, Switzerland) and lyophilized using a cryodos freeze dryer (Telstar, Barcelona, Spain) to yield 26.67 g of solid extract (1.28% W/W). These steps ensure complete evaporation of ethanol, leaving behind a solid mass [15].

### 2.3. Phytochemical Screening of* V. vinifera* Seed

The phytochemical composition of* V. vinifera* seeds was screened by using a standard method as described by Harborne [16]. The compounds analyzed include alkaloids, proteins, glycosides, tannin, steroids, phenol, lignins, saponins, monoterpenoids, flavonoids, carbohydrates, oils, and fats.

### 2.4. Animals

Male albino rats of Wistar strain with body weight between 175 to 200 g were procured from Animal House, Faculty of Medicine, University of Malaya, Kuala Lumpur, Malaysia. The animals were maintained at room temperature of 25±2°C and 12/12 hr light/dark cycle. Animals were given standard commercial rat chow diet (Harlan, UK) and tap water* ad libitum*. Experimental procedures were in accordance with ARRIVE guidelines (Animals in Research: Reporting In-Vivo Experiments) and European Community Guidelines/EEC Directive, 1986. This study was approved by the Faculty of Medicine, Animal Care and Use Committee, with ethics number: FIS/01/12/2013. Acute toxicity study was conducted according to Organization for Economic Cooperation and Development (OECD) revised up-and-down procedure for acute toxicity testing (OECD guideline 425) [17]. Thirty male Wistar rats were divided into five groups with each group received a single dose of 100, 500, 1000, and 3000 mg/kg bw of* V. vinifera* seed ethanolic extract. No signs of toxicity were observed at these tested doses.

### 2.5. Induction of Diabetes

Overnight (12 h) fasted animals were rendered diabetes via a single intraperitoneal (i.p) injection of a freshly prepared STZ (55 mg/kg bw) dissolved in 0.1 M citrate buffer (pH 4.5). STZ injected animals were given 5% glucose solution for 24 hr to overcome drug-induced hypoglycemia. Diabetes was confirmed by the presence of polydipsia, polyuria, and weight loss and only animals exhibiting fasting blood glucose (FBG) levels between 300–400 mg/dL three days following STZ injection were used [18]. Treatment was commenced on the fourth day of STZ injection which was considered as day one.* V. vinifera* seed ethanolic extract was administered orally at 250 and 500 mg/kg/day according to the previously reported doses [11], in a form of suspension in 1% sodium carboxy methyl cellulose (Na-CMC) in distilled water. The extract was administered by using oral gavage tube daily for 28 consecutive days.

### 2.6. Experimental Design

Rats were divided into five groups with six animals per group as follows: Group I, control rats, received 1% Na-CMC vehicle only; Group II, diabetic control rats, received 1% Na-CMC vehicle only; Group III, diabetic rats, was treated with* V. vinifera* seed ethanolic extract at 250 mg/kg bw; Group IV, diabetic rats, was treated with* V. vinifera* seed ethanolic extract at 500 mg/kg bw; and Group V, diabetic rats, was treated with standard drug, glibenclamide at 600 *μ*g/kg bw as previously described [19].

At the end of 28-day treatment, animals were fasted overnight prior to sacrificed. Immediately after sacrificed, the liver was excised and was then stored at −80°C for later analysis or immediately used. In the meantime, blood was withdrawn via direct heart puncture and was then stored into tubes for serum analyses of total protein, total bilirubin, ALT, AST, ACP, ALP, and GGT levels. Serum and liver homogenates were analyzed for the presence of ethyl glucoronide (EtG), a biomarker for ethanol consumption [20], using ELISA kit (Microgenics Corp., Thermo Fisher Scientific, Fremont, CA, USA). In all samples, no traces of EtG were detected.

### 2.7. Preparation of Liver Mitochondrial and Cytosolic Fractions

Liver was weighed and 10% tissue homogenate was prepared in phosphate buffer (0.1 M, pH 7.4) using a glass-Teflon homogenizer (Heidolph Silent Crusher M, Germany). Homogenates were centrifuged at 500 g at 4°C for 10 min. Supernatant was collected and recentrifuged at 2000 g for 10 min. Supernatant was again collected and recentrifuged at 12,000 g at 4°C for 10 min, and pellet was resuspended in 200 mM mannitol, 50 mM sucrose, 10 mmol/L Hepes-KOH (pH 7.4) and stored as mitochondrial fraction at −80°C. The final supernatant was taken and centrifuged for 1 hr at 40,000 g [21]. The resulting supernatant was used as cytosolic fraction and was stored at 4°C. In the present study, mitochondrial fraction was used to determine ICDH, SDH, and MDH enzymes activity levels while cytosolic fraction was used to determine LDH, G-6-PDH, ALT, and AST enzymes activity levels.

### 2.8. Estimation of Carbohydrate Metabolizing Enzymes Activity Levels

LDH (EC: 1.1.1.27) activity levels were measured following the method of Srikantan and Krishnamurti [22]. ICDH (EC: 1.1.1.41) activity levels were estimated according to the method of Kornberg and Pricer [23]. SDH (EC: 1.3.99.1) and MDH (EC: 1.1.1.37) activity levels were estimated according to the method of Nachlas et al. [24] while G-6-PDH (EC: 1.1.1.49) activity levels were measured according to the method of Bergmeyer and Bernt [25]. Enzyme activity levels were expressed as *μ*mol of formazan formed/mg protein/hr.

### 2.9. Estimation of Liver Enzymes Levels in Serum and Liver Homogenates

The levels of AST (EC: 2.6.1.1) and ALT (EC: 2.6.1.2) in liver homogenates were estimated according to the method of Bergmeyer and Bernt [25]. Meanwhile, serum levels of AST, ALT, ALP, and total bilirubin were estimated according to the protocol of the manual of diagnostic kits (Randox Laboratories Ltd, Crumlin, UK).

### 2.10. Histopathological Changes of the Liver

Liver was excised immediately following sacrifice, washed with a phosphate buffer solution, and then fixed in 10% formalin. Tissues were dehydrated through graded series of alcohol, cleared in xylene, and embedded in paraffin wax. Tissues were then cut into sections of 5 *μ*m in thickness using a microtome (Histo-line laboratories, ARM-3600, Viabrembo, Milano, Italy) and stained with hematoxylin-eosin (H&E). Histopathological changes were examined under phase contrast microscope (Nikon H600L, Japan) and images were captured at magnification of 40× using a computer-assisted image analyzer (Nikon H600L, Nikon DS camera control Unit DS-U2, Version 4.4). Histopathological changes such as necrosis, sinusoidal hyperemia, and connective tissue inflammation of the portal region were scored by three independent observers following the description by Guven et al. [26] (0: normal, no changes, +: mild, ++: moderate, and +++: severe changes).

### 2.11. Estimation of TBARS Levels in Liver Homogenates

TBARS measures the malondialdehyde (MDA) levels, a lipid peroxidation (LPO) product present in the sample. Determination was made according to the method of Esterbauer and Cheeseman [27]. The rate of lipid peroxidation was expressed as *μ*moles of MDA formed/gram wet weight of tissue.

### 2.12. Statistical Analysis

The values were expressed as mean ± standard deviation (SD) (*n* = 6). Statistical analyses were performed by one way analysis of variance (ANOVA) and Student's *t*-test followed by* post hoc* statistical test. Significant difference was analyzed at *P* level <0.05.

## 3. Results 

### 3.1. Phytochemical Screening

Preliminary phytochemical screening of the seed extract of* V. vinifera* revealed the presence of alkaloids, flavonoids, glycosides, saponins, steroids, lignins, phenols, tannins, and monoterpenoids (data was not shown).

### 3.2. Effects of* V. vinifera *Seed Ethanolic Extract on Liver LDH, ICDH, SDH, MDH, and G-6-PDH Activity Levels

Table 1 shows the effect of* V. vinifera *seed ethanolic extract on activity levels of liver carbohydrate metabolizing enzymes in different experimental groups. Our findings indicate that SDH activity was the highest followed by G-6-PDH, LDH, ICDH, and MDH. In diabetic rats, activity levels of ICDH, SDH, MDH, and G-6-PDH were significantly decreased while LDH activity level was markedly increased as compared to normal, nondiabetic rats. Administration of 250 mg/kg/day and 500 mg/kg/day* V. vinifera* seed ethanolic extract resulted in a significantly higher ICDH, SDH, MDH, and G-6-PDH activity levels and lower LDH activity levels as compared to nontreated diabetic rats. 500 mg/kg/day* V. vinifera* seed ethanolic extract had an almost similar potency to glibenclamide in preventing the changes in LDH, SDH, G-6-PDH, ICDH, and MDH activity levels in diabetic rats liver.

### 3.3. Effects of* V. vinifera* Seed Ethanolic Extract on Serum Levels of ALT, AST, ALP, ACP, and GGT

Table 2 shows the effect of* V. vinifera* seed extract or glibenclamide on serum ALT, AST, ALP, ACP, and GGT in different experimental groups. In nontreated diabetic rats, the levels of ALT, AST, ALP, ACP, and GGT were significantly higher than normal, nondiabetic rats. Treatment with 250 mg/kg/day and 500 mg/kg/day of the seed extract or glibenclamide resulted in lower serum level of these enzymes as compared to nontreated diabetic rats. 500 mg/kg/day* V. vinifera* seed had lesser effect than glibenclamide in preventing the increase in serum ALT, AST, ALP, and ACP levels in diabetic rats.

### 3.4. Effects of* V. vinifera* Seed Ethanolic Extract on Serum Levels of Bilirubin and Total Protein

Table 2 shows changes in total protein and bilirubin levels in the serum of different experimental groups. Our findings indicate that total protein levels were lower while total bilirubin levels were markedly higher in diabetic rats as compared to normal, nondiabetic rats. Treatment with 250 mg/kg/day and 500 mg/kg/day* V. vinifera* seed extract or glibenclamide resulted in higher total protein but lower total bilirubin levels as compared to nontreated diabetic rats. 500 mg/kg/day* V. vinifera* seed extract had lesser effect than glibenclamide in preventing the decrease in total protein and the increase in total bilirubin levels in the liver of diabetic rats.

### 3.5. Effect of* V. vinifera* Seed Ethanolic Extract on Liver ALT and AST Levels

Table 2 shows the levels of ALT and AST in liver homogenates of different experimental groups. Our findings indicate that the levels of these enzymes were markedly increased in diabetic rats as compared to normal, nondiabetic rats. Treatment with 250 mg/kg/day and 500 mg/kg/day* V. vinifera *seed extract or glibenclamide resulted in lower ALT and AST levels as compared to nontreated diabetic rats. 500 mg/kg/day* V. vinifera *seed produced a slightly lesser effect than glibenclamide in preventing the increase in ALT and AST levels of the liver of diabetic rats.

### 3.6. Effect of* V. vinifera *Seed Extract on Histopathological Changes of the Liver

**Figure 1:**
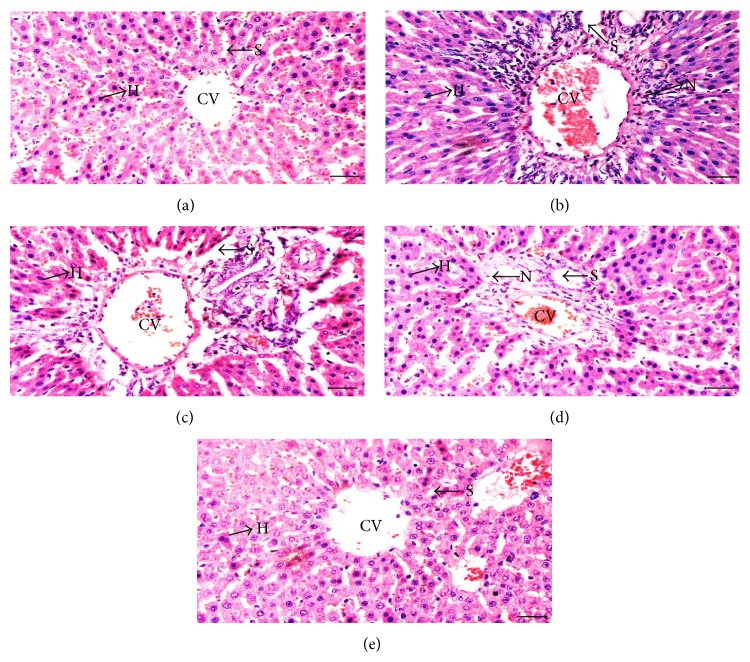
Effect of the seed ethanolic extract of* V. vinifera *on liver histology. Representative images of the liver in (a) normal, (b) STZ-induced diabetic rats, (c) diabetic rats treated with 250 mg/kg/day* V. vinifera* seed extract, (d) diabetic rats treated with 500 mg/kg/day* V. vinifera* seed extract, and (e) diabetic rats treated with 600 *μ*g/kg/day glibenclamide. Images were taken under 20× magnification. Scale bar represents 50 *μ*m. In diabetic rats, several areas of moderate to severe necrosis could be seen around the central vein. Mild to absence of necrotic changes could be seen following treatment with 500 mg/kg/day* V. vinifera* or glibenclamide to diabetic rats. H = hepatocytes, CV = central vein, S = sinusoid, and N = necrosis (40× magnification).

Figure 1 shows histopathological changes while Table 3 shows semiquantitative analyses of inflammatory changes in the liver of diabetic rats receiving* V. vinifera *seed extract or glibenclamide treatment. In normal, nondiabetic rats, a distinct and well-arranged hepatocytes, sinusoids, and central vein could be seen (Figure 1(a)). Meanwhile, in diabetic rats, hepatocytes were disorganized with several areas of necrosis. Sinusoids were enlarged with the wall of veins thickened (Figure 1(b)).* V. vinifera *seed extract or glibenclamide treatments prevented these changes as evidenced by lesser signs of necrosis, lack of central hemorrhagic necrosis, mild sinusoid hyperemia, and mild connective tissue inflammation in the portal region (Figures 1(c), 1(d), and 1(e)).

### 3.7. TBARS Levels in Liver Homogenates

**Figure 2:**
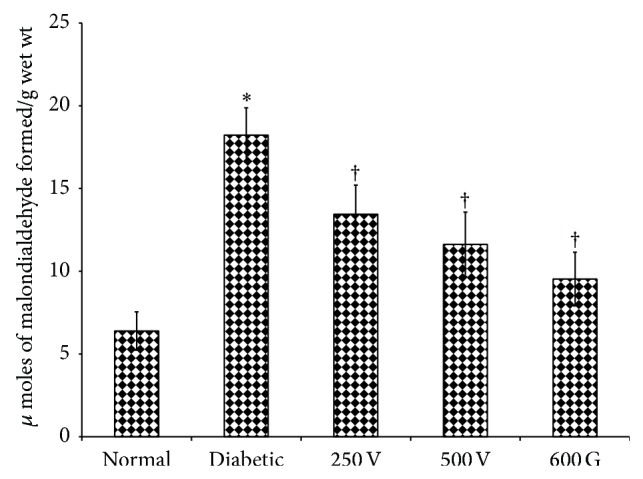
Effect of seed ethanolic extract of* V. vinifera *on TBARS levels. A significantly higher liver TBARS levels were observed in diabetic rats as compared to normal, nondiabetic rats. Administration of glibenclamide or seed ethanolic extract of* V. vinifera *prevented the increase in TBARS levels in diabetic rats. 250 V: 250 mg/kg/day* V. vinifera* seed extract; 500 V; 500 mg/kg/day* V. vinifera* seed extract; 600 G: 600 *μ*g/kg/day glibenclamide. *n* = 6, ^*^ *P* < 0.05 as compared to normal, nondiabetic rats, and ^†^ *P* < 0.05 as compared to nontreated diabetic rats.

In Figure 2, TBARS levels in liver homogenates in nontreated diabetic rats liver were markedly higher as compared to normal, nondiabetic control rats (*P* < 0.05). Administration of 250 and 500 mg/kg* V. vinifera *seed extract or glibenclamide resulted in a significant decrease in the level of liver TBARS as compared to nontreated diabetic rats.

## 4. Discussion 

Chronic hyperglycemia and insulin deficiency can produce various disruptions to the metabolic processes in the liver. Additionally, diabetes has also been reported to cause liver damage [28]. In the present study, orally administered ethanolic seed extract of* V. vinifera *from Muscat variety to diabetic rats was able to prevent the decrease in activity levels of key enzymes involved in liver carbohydrate metabolism which include the G-6PDH, ICDH, SDH, and MDH. We have shown that administration of the seed extract to diabetic rats prevented hepatocyte destruction as evidenced from near normal serum levels of ALT, AST, ALP, ACP, GGT, and total bilirubin. The total protein level in the serum was also maintained near normal following supplementation with the seed extract.

In this study, activity levels of liver mitochondrial enzymes (ICDH, SDH, and MDH) were markedly reduced in diabetes. These enzymes are involved in ATP generation which yielded 36 moles of ATPs per mole of glucose [3]. SDH and MDH are the two Krebs cycle enzymes where the former has the highest activity as compared to other enzymes in the cycle [29]. In diabetes, activity of Krebs cycle enzymes was lower than normal [30, 31], resulting in impairment of ATP generation. These may compromise the liver biosynthetic, degradation, and detoxification functions. However, despite of diabetes-induced decrease in activity levels of liver Kreb cycle enzymes, LDH activity level was markedly increased. Similar findings were reported by others [29, 32]. LDH is the terminal glycolytic enzyme involved in pyruvate interconversion to lactate to produce energy under anaerobic condition [3]. The significance of LDH increase in diabetes is unknown; however this could be related to lower amount of insulin as insulin has been reported to affect the activity of LDH [33]. Recent evidence indicated that increased in cellular activity of LDH in diabetes was due to increase in peroxide (H_2_O_2_) levels [34]. Ability of the seed extract to lower the free radical levels in diabetes could explain the decrease in hepatic LDH activity levels; however this needs to be confirmed. In our study, lower activity levels of G-6-PDH were observed in the liver of diabetic rats which was consistent with other findings [29, 35]. G-6-PDH is a highly specific enzyme involved in NADPH generation in the pentose phosphate pathway [2]. Activity levels of pentose phosphate and glycolytic pathways enzymes were reported to decrease in experimental diabetic animals [36]. Besides liver, PPP enzymes activity levels in the brain were also reported to reduce in STZ-induced diabetic rats [37].

This study has provided evidences of hepatocyte damage from elevated levels of liver enzymes (ALT, AST, ACP, ALP, and GGT) in serum and liver homogenates in diabetes. The increase in serum levels of ALT and AST indicates hepatocellular injury where these enzymes were released into the circulation while the elevated levels of serum GGT and ALP indicates biliary tree obstruction most likely due to edematous compression. The serum levels of total bilirubin were also increased indicating either intra- or extrahepatic biliary tree obstruction. These findings were consistent with others who reported the rise in serum ALT [38], AST [39], ACP [40], GGT, and AST [41] in diabetic rodents and humans. In this study, the levels of AST and ALT were significantly higher than ALP consistent with the reports of a highly elevated ALT level in patient with type 2 diabetes [9].

The deranged liver function test (LFT) parameters in both serum and liver homogenates in diabetic rats indicative of hepatocellular damage were supported by histopathological changes of the liver as featured by moderate to severe necrosis. The structural changes might cause compression of the biliary trees, resulting in the rise in serum levels of ALP, GGT, and total bilirubin in diabetic animals. Administration of* V. vinifera* seed prevented the histopathological changes in the liver as well as lowered the serum levels of liver enzymes and total bilirubin. Lesser signs of necrosis, hyperemia, and connective tissue inflammation were seen in the liver of* V. vinifera*-treated diabetic rats. An elevated level of serum total proteins following* V. vinifera *seed extract treatment to diabetic rats suggested that function of the liver was restored to near normal following an insult caused by diabetes.

Our findings indicated that TBARS levels in the liver homogenates were reduced following* V. vinifera *seed ethanolic extract administration to diabetic rats. Previous study has shown that the red grape seed (*V. vinifera* variety Burgund mare) reduces the oxidative stress level in diabetic rats as evidence from the decrease in TBARS levels [42]. Phytochemical screening showed that the seed extract contains flavonoids and phenols, the two compounds known to possess antioxidant activities [43]. Therefore, the ethanolic extract of* V. vinifera *seed could help to reduce the levels of oxidative stress in the liver of diabetic rats most likely via scavenging the free radicals that are highly elevated in diabetes [44]. In addition, flavonoid may help to improve the activity of carbohydrate metabolizing enzymes in the liver from an observation that high flavonoid-rich green tea improves the activity of hepatic carbohydrate metabolizing enzymes [1]. Further works are needed to better characterize the active compounds present in* V. vinifera* seed that are responsible for reducing the oxidative stress level in diabetes and to identify the levels of endogenous antioxidant enzymes following seed extract administration. Additionally,* in vitro* antioxidant assay of the seed extract will also be carried out in order to support its claimed free radicals scavenging activity.

## 5. Conclusions 

Our study has shown for the first time the effect of ethanolic seed extract of Muscat variety of* V. vinifera *against deterioration of activity levels of key enzymes involved in liver carbohydrate metabolism in diabetes. The seed extract helps to prevent liver damage due to oxidative stress which may contribute towards improvement in liver function and histology. Further study will include identifying the effect of the seed extract on other key carbohydrate metabolizing enzymes such as hexokinase, pyruvate kinase, glycogen synthase, and phosphorylase. Taken into account various limitations, this study provides preliminary evidence that the seed extract of a Muscat variety of* V. vinifera* helps in liver protection in diabetes.

## Figures and Tables

**Table 1:** Effect of *V. vinifera* seed ethanolic extract on liver SDH, ICDH, MDH, LDH, and G-6-PPDH in streptozotocin-induced diabetic rats.

Parameters	Normal	Diabetic	Diabetic		
			250 mg/kg *V. vinifera *	500 mg/kg *V. vinifera *	600 *μ*g/kg glibenclamide
SDH^#^	4.51 ± 0.08	2.15^*^ ± 0.05	2.93^†^ ± 0.06	3.29^†^ ± 0.08	2.84^†^ ± 0.07
ICDH^#^	0.78 ± 0.04	0.4^*^ ± 0.04	0.57^†^ ± 0.06	0.65^†^ ± 0.07	0.68^†^ ± 0.06
MDH^#^	0.62 ± 0.08	0.32^*^ ± 0.08	0.48^†^ ± 0.06	0.53^†^ ± 0.08	0.56^†^ ± 0.09
LDH^#^	0.94 ± 0.06	1.75^*^ ± 0.08	1.26^†^ ± 0.08	1.06^†^ ± 0.07	1.18^†^ ± 0.06
G-6-PDH^#^	1.78 ± 0.04	0.73^*^ ± 0.06	1.27^†^ ± 0.08	1.44^†^ ± 0.05	1.38^†^ ± 0.06

^#^(*μ* moles of formazan formed/mg protein/h). Value represents mean ± SD for 6 rats per group. ^*^ *P* < 0.05 as compared to normal, nondiabetic rats group and ^†^ *P* < 0.05 as compared to nontreated diabetic rats.

**Table 2:** Effect of *V. vinifera* seed ethanolic extract on serum ALT, AST, ALP, ACP, GGT, total protein, total bilirubin, and liver ALT and AST in streptozotocin-induced diabetic rats.

Parameters	Normal	Diabetic	Diabetic		
			250 mg/kg *V. vinifera *	500 mg/kg *V. vinifera *	600 *μ*g/kg glibenclamide
Serum					
ALT (U/L)	142.39 ± 6.32	236.18^*^ ± 15.09	213.76^†^ ± 9.18	184.13^†^ ± 11.37	169.37^†^ ± 9.43
AST (U/L)	103.78 ± 8.64	184.67^*^ ± 7.15	145.91^†^ ± 9.49	139.36^†^ ± 9.62	125.17^†^ ± 10.23
ALP (U/L)	46.75 ± 3.65	247.25^*^ ± 8.46	166.92^†^ ± 5.69	132.54^†^ ± 3.73	112.73^†^ ± 4.67
ACP (U/L)	11.54 ± 0.58	21.58^*^ ± 0.84	18.64^†^ ± 0.94	14.52^†^ ± 0.87	12.68^†^ ± 0.76
GGT (U/L)	10.23 ± 0.72	14.75^*^ ± 0.14	8.65^†^ ± 0.76	8.45^†^ ± 0.89	8.79^†^ ± 0.02
Total protein (U/L)	8.96 ± 0.75	4.22^*^ ± 0.86	5.26^†^ ± 0.34	7.45^†^ ± 0.86	7.84^†^ ± 0.73
Total bilirubin (U/L)	0.43 ± 0.05	4.58^*^ ± 0.05	2.33^†^ ± 0.06	1.58^†^ ± 0.04	1.16^†^ ± 0.05
Liver tissue					
ALT (*μ* moles of pyruvate formed/mg protein/h)	0.48 ± 0.08	0.78^*^ ± 0.12	0.57^†^ ± 0.13	0.52^†^ ± 0.11	0.48^†^ ± 0.09
AST (*μ* moles of pyruvate formed/mg protein/h)	0.37 ± 0.06	0.65^*^ ± 0.09	0.56^†^ ± 0.05	0.43^†^ ± 0.09	0.36^†^ ± 0.06

Value represents mean ± SD for 6 rats per group.

^*^ *P* < 0.05 as compared to normal, nondiabetic rats group and ^†^ *P* < 0.05 as compared to nontreated diabetic rats.

**Table 3:** Semiquantitative analyses of histopathological changes of the liver.

Parameters	Normal	Diabetic	Diabetic		
			250 mg/kg *V. vinifera *	500 mg/kg *V. vinifera *	600 *μ*g/kg glibenclamide
Necrosis	0	++	+	+	+
Sinusoidal hyperemia	0	++	++	+	0
Connective tissue inflammation in portal region	0	++	+	0	0

0: no changes, +: mild changes, ++: moderate changes, and +++: severe changes.
